# Prevalence and Predictors of Symptoms of Anxiety or Depression at Diagnosis in Patients With Inflammatory Bowel Disease: An Inception Cohort

**DOI:** 10.1111/apt.70248

**Published:** 2025-06-29

**Authors:** Christy Riggott, David J. Gracie, Alexander C. Ford

**Affiliations:** ^1^ Leeds Gastroenterology Institute St. James's University Hospital Leeds UK; ^2^ Leeds Institute of Medical Research at St. James's University of Leeds Leeds UK

**Keywords:** gut‐brain axis, inception cohort, inflammatory bowel disease, psychology

## Abstract

**Background:**

The prevalence of symptoms of a common mental disorder, including anxiety or depression, is high among patients with established inflammatory bowel disease (IBD). This may represent a therapeutic target for affected patients. However, whether these symptoms arise from genuine gut‐brain effects, or are merely a consequence of a preceding adverse disease course is unclear.

**Aims:**

To assess prevalence and predictors of anxiety and depression in an inception cohort of patients with IBD.

**Methods:**

We collected demographic data, disease‐related information, diagnosis of a pre‐existing common mental disorder, symptoms of a common mental disorder, using the hospital anxiety and depression score, and gastrointestinal symptom‐specific anxiety, using the Visceral Sensitivity Index (VSI), from individuals newly diagnosed with IBD during their index outpatient appointment or inpatient admission. The prevalence of symptoms of a common mental disorder at diagnosis, and predictors of the presence of these symptoms, were examined.

**Results:**

Of 300 participants, 117 (39.0%) reported symptoms of a common mental disorder (107 (35.7%) anxiety, 47 (15.7%) depression). Younger age, female sex, tobacco use, a longer duration of symptoms prior to diagnosis, higher gastrointestinal symptom‐specific anxiety, and stressful life events in the preceding 12 months were associated with a significantly increased likelihood of reporting these symptoms. Higher gastrointestinal symptom‐specific anxiety remained significant following logistic regression (OR 2.19; 95% CI 1.00–4.79 for VSI moderate and OR 13.5; 95% CI 5.86–31.2 for VSI high, *p* < 0.001 for trend).

**Conclusion:**

Poor psychological health is highly prevalent at the time of an IBD diagnosis, suggesting genuine gut‐brain effects.

## Graphical abstract

Poor psychological health is highly prevalent at the time of an IBD diagnosis and is associated with gastrointestinal symptom‐specific anxiety and the occurrence of stressful life events in the 12 months prior to diagnosis. These findings suggest genuine gut‐brain effects in IBD.

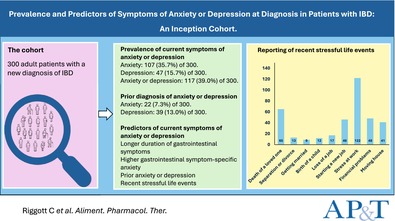

## 1 Introduction

Crohn's disease (CD) and ulcerative colitis (UC) are chronic relapsing–remitting immune‐mediated inflammatory bowel diseases (IBD) affecting over 8 million individuals worldwide [1]. Mucosal inflammation is the hallmark of disease activity and a presumed consequence of immune dysregulation in response to environmental and microbiome triggers in genetically susceptible individuals [2]. Gastrointestinal inflammatory activity produces troublesome symptoms, including abdominal pain, diarrhoea and faecal urgency, which are associated with a reduced quality of life, the development of functional disability and, ultimately, progressive digestive damage with resulting disease complications. Therefore, current treatment paradigms focus on proactive treatment of mucosal inflammation [3, 4, 5, 6, 7].

The gut‐brain axis may also exert an influence on the natural history of IBD [8, 9]. The prevalence of symptoms of a common mental disorder, such as anxiety or depression, is more than double that of the general population, and peaks during periods of disease activity, suggesting a possible link with inflammation [8, 9, 10, 11]. Furthermore, symptoms of a common mental disorder in individuals with IBD are associated with future adverse disease outcomes, including flare of disease activity, treatment escalation, hospitalisation and intestinal resection, in addition to increased healthcare utilisation and increased healthcare costs, compared with individuals without such symptoms [8, 11, 12, 13, 14, 15]. Such findings suggest that, alongside managing co‐existing inflammatory burden, the gut‐brain axis may be a therapeutic target in IBD [9, 16, 17].

To examine whether the high prevalence of symptoms of anxiety and depression among patients with IBD is mainly a consequence of a pre‐existing adverse disease course, such as repeated hospitalisation or intestinal resection, studies assessing the reporting of these symptoms in patients with newly diagnosed IBD, and before the onset of disease‐related complications, are essential. If such studies determine poor psychological health to be highly prevalent in this group of patients, it would support a move towards a biopsychosocial model of care [18], where integrated psychological input may serve to mitigate future disease complications. This is of particular relevance as gut‐brain axis directed therapies, including psychological therapies and gut‐brain neuromodulators, have demonstrated a benefit in patients with IBD with evidence of poor psychological health [19, 20].

To date, however, there have been few studies reporting the prevalence of symptoms of a common mental disorder in patients with newly diagnosed IBD. In a Spanish study of 156 patients, the authors reported a 37% prevalence of symptoms of anxiety and a 17% prevalence of symptoms of depression in 155 patients with a recent diagnosis of IBD [21]. These rates are comparable to those in patients with established disease [10]. However, this study recruited participants whom had received the diagnosis of IBD within the preceding 6 months, and failed to control for any adverse disease outcomes occurring during the time since diagnosis, but prior to study recruitment [21]. This could be a confounding factor, particularly as hospitalisation and surgery have the potential to negatively impact subsequent psychological health [22]. In another study from New Zealand, which included data from 53 newly diagnosed patients with IBD, abnormal anxiety scores were reported by 23%, and abnormal depression scores by 9% [23].

We, therefore, examined the prevalence of symptoms of anxiety and depression, and associated factors, in a large inception cohort of patients with IBD, with the hypothesis that these symptoms are already highly prevalent at the time of diagnosis.

## 2 Methods

### 2.1 Participants and Setting

Consecutive adult patients ≥ 18 years old with a new diagnosis of CD, UC or IBD unclassified (IBD‐U) based on endoscopic, histological, or radiological findings were invited to participate in this prospective cross‐sectional survey conducted in Leeds Teaching Hospitals NHS Trust between March 2023 and January 2025. Participation in the study required the completion of a baseline questionnaire and, therefore, patients who were unable to understand written English were excluded. Furthermore, as surgery may independently influence psychological health [22], patients who underwent emergency surgery for IBD during their index presentation, prior to enrolment in the study, were excluded.

Individuals were identified by an individual assessor (CR) by performing a weekly review of IBD clinic patient lists and inpatient admission sheets against the study eligibility criteria. Following this, participants deemed by the assessing clinician to have an alternate pathology, including infective, ischaemic, drug‐induced, or diverticular‐associated colitis were excluded. Participants with non‐specific terminal ileitis, in whom there was insufficient histological or radiological evidence to confirm a formal diagnosis of IBD, were excluded from participation until a definitive diagnosis was reached.

Eligible individuals were recruited in person, at their index clinic consultation or during their index hospital admission. This study was approved by the Wales Research and Ethics Committee (REC ref.: 22/WA/0368).

### 2.2 Data Collection and Synthesis

The date of study recruitment, type of IBD, including disease extent, location and phenotype, and IBD‐related medications commenced at diagnosis (5‐aminosalicylates, immunomodulators, such as azathioprine or mercaptopurine, corticosteroids, or advanced therapies, including biologics and small molecules) were recorded at baseline, in addition to demographic data, including age, sex and lifestyle factors, and the presence of an existing formal diagnosis of either anxiety or depression at baseline. Furthermore, we collected data regarding total duration of gastrointestinal symptoms prior to the diagnosis of IBD being made, and the occurrence of any potentially stressful life events in the 12 months prior to diagnosis, both by self‐report. These included the death of a loved one, the birth of a child, separation or divorce, getting married, loss of a job, commencing a new job, stress at work, financial problems or moving to a new home.

We collected data on gastrointestinal symptom‐specific anxiety, which is a cumulative cognitive and behavioural response arising from fear of gastrointestinal symptoms. For this purpose, we used the Visceral Sensitivity Index (VSI), which is a 15‐item scale with scores for each item ranging from 0 to 6, and a total possible score of 90 [24]. As there are currently no validated cut‐offs to define low, medium, or high levels of gastrointestinal symptom‐specific anxiety we divided total VSI scores into three evenly distributed tertiles, representing low, medium, or high levels of gastrointestinal symptom‐specific anxiety respectively.

The presence of symptoms suggestive of anxiety or depression was assessed using the 14‐item hospital anxiety and depression scale (HADS), with scores for each item ranging from 0 to 3, and a total score of 21 for either anxiety or depression [25]. A score of 0–7 is considered normal, 8–10 borderline abnormal, and ≥ 11 abnormal, according to the original validation study. We, therefore, used a cut‐off of ≥ 11 to define the presence of symptoms of either anxiety or depression in this study.

All participants had undergone investigation for gastrointestinal symptoms, with endoscopic, histologic, or radiologic assessment demonstrating active mucosal inflammation to confirm the diagnosis of IBD. Hence, disease activity questionnaires were not administered at baseline as, essentially, all enrolled patients had mucosal disease activity around the time of recruitment. Participants with IBD‐U and UC were considered collectively for all analyses.

### 2.3 Statistical Analysis

We compared baseline characteristics of participants, including demographics, disease extent, location and phenotype, IBD‐related medications, and levels of gastrointestinal symptom‐specific anxiety, according to the presence or absence of symptoms compatible with anxiety or depression at baseline using a *χ* ^2^ test for categorical variables and an independent samples *t*‐test for continuous data. Due to multiple comparisons, a 2‐tailed *p* value of < 0.01 was considered statistically significant for all analyses. To determine factors independently associated with the reporting of symptoms of a common mental disorder, we performed logistic regression, controlling for all baseline demographic data, in addition to gastrointestinal symptom‐specific anxiety, the presence of stressful life events in the year prior to diagnosis, and the duration of symptoms prior to diagnosis. These were reported using odds ratios (OR) with 95% confidence intervals (CI). We used SPSS for Windows version 29.0 (SPSS Inc., Chicago, IL, USA) to perform all statistical analyses.

## 3 Results

**FIGURE 1:**
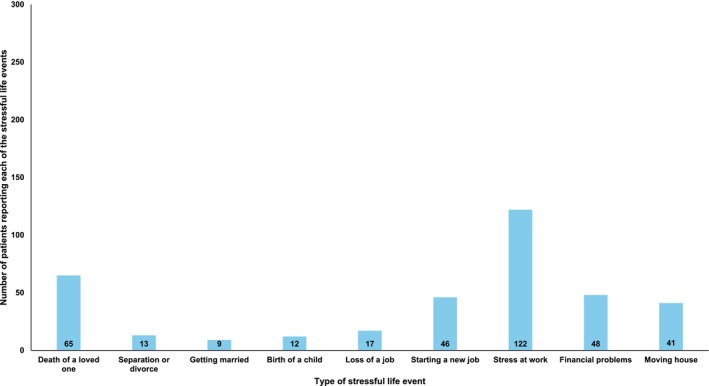
Number of each of the stressful life events of interest in the 12 months prior to diagnosis among 300 patients with newly diagnosed IBD at baseline.

A total of 309 patients were approached to participate in the study, of whom 300 consented (97.1%). Of these, 282 (94.0%) were recruited in outpatient clinics, and the remaining [26] were recruited during an index inpatient admission. The mean duration from diagnosis to recruitment was 7 days. Data capture at baseline was complete for all 300 participants (mean age at baseline 41.6 years (range 18–87 years), 142 (47.3%) female, 99 (33.0%) CD, 162 (54.0%) UC and 39 (13.0%) IBD‐U, 249 (83.0%) White) (Table 1). In total, 264 (88.0%) patients were commenced on an IBD‐related medication at the time they were recruited to the study. The number of patients reporting each of the stressful life events of interest in the 12 months prior to diagnosis is reported in Figure 1.

**TABLE 1:** Characteristics of 300 patients with newly diagnosed IBD according to the presence or absence of symptoms of anxiety or depression at baseline.

	All patients (*n* = 300)	Patients with symptoms of anxiety at baseline (*n* = 107)	Patients without symptoms of anxiety at baseline (*n* = 193)	*p* *	Patients with symptoms of depression at baseline (*n* = 47)	Patients without symptoms of depression at baseline (*n* = 253)	*p* *	Patients with symptoms of anxiety or depression at baseline (*n* = 117)	Patients without symptoms of anxiety or depression at baseline (*n* = 183)	*p* *
Mean age in years (SD)	41.6 (16.2)	37.3 (13.5)	43.9 (17.1)	< 0.001	37.7 (13.7)	42.3 (16.5)	0.078	37.6 (13.9)	44.1 (17.0)	< 0.001
Female sex (%)	142 (47.3)	64 (59.8)	78 (40.4)	< 0.001	24 (51.1)	118 (46.6)	0.58	68 (58.1)	74 (40.4)	0.003
Married or co‐habiting (%)	170 (56.7)	53 (49.5)	117 (60.6)	0.063	21 (44.7)	149 (58.9)	0.071	58 (49.6)	112 (61.2)	0.05
White Caucasian (%)	249 (83.0)	93 (86.9)	156 (80.8)	0.18	37 (78.7)	212 (83.8)	0.40	101 (86.3)	148 (80.9)	0.22
University graduate/professional (%)	103 (34.3)	19 (17.8)	31 (16.1)	0.71	5 (10.6)	45 (17.8)	0.23	37 (31.6)	66 (36.1)	0.43
Tobacco user (%)	45 (15.0)	24 (22.4)	21 (10.9)	0.007	13 (27.7)	32 (12.6)	0.008	26 (22.2)	19 (10.4)	0.005
Alcohol user (%)	186 (62.0)	63 (58.9)	123 (63.7)	0.41	21 (44.7)	165 (74.0)	0.008	67 (57.3)	119 (65.0)	0.18
Outpatient diagnosis (%)	282 (94.0)	101 (94.4)	181 (93.8)	0.83	43 (91.5)	239 (94.5)	0.43	108 (92.3)	174 (95.1)	0.32
IBD type (%)										
CD	99 (33.0)	41 (38.3)	58 (30.1)		16 (34.0)	83 (32.8)		43 (36.8)	56 (30.6)	0.53
UC	162 (54.0)	53 (49.5)	109 (56.5)		26 (55.3)	136 (53.8)		59 (50.4)	103 (56.2)	
IBD‐U	39 (13.0)	13 (12.1)	26 (13.5)	0.34	5 (10.6)	34 (13.4)	0.87	15 (12.8)	24 (13.1)	
Commenced 5‐aminosalicylate (%)	179 (59.7)	55 (51.4)	124 (64.2)	0.030	24 (51.1)	155 (61.3)	0.19	61 (51.2)	118 (64.5)	0.034
Commenced immunomodulator (%)	13 (4.3)	5 (4.7)	5 (2.6)	0.83	4 (8.5)	9 (3.6)	0.13	7 (6.0)	6 (3.3)	0.26
Commenced advanced therapy (%)	39 (13.0)	18 (16.8)	21 (10.9)	0.14	10 (21.3)	29 (11.5)	0.066	20 (17.1)	19 (10.4)	0.092
Commenced glucocorticosteroids (%)	95 (31.7)	40 (37.4)	55 (28.5)	0.11	18 (38.3)	77 (30.4)	0.29	43 (36.8)	52 (28.4)	0.13
Commenced any IBD‐related medication (%)	264 (88.0)	93 (86.9)	171 (88.6)	0.68	42 (89.4)	222 (87.7)	0.75	102 (87.2)	162 (88.5)	0.73
Level of gastrointestinal symptom‐specific anxiety on VSI										
Low	100 (33.3)	12 (11.2)	88 (45.6)		6 (12.8)	94 (37.2)		15 (12.8)	85 (46.4)	< 0.001
Moderate	100 (33.3)	30 (28.0)	70 (36.3)		6 (12.8)	94 (37.2)		31 (26.4)	69 (37.7)	
High	100 (33.3)	65 (60.7)	35 (18.1)	< 0.001	35 (74.5)	65 (25.7)	< 0.001	71 (60.7)	29 (15.8)	
One or more stressful life events in the preceding 12 months (%)	197 (65.7)	81 (75.7)	116 (60.1)	0.006	39 (83.0)	158 (62.5)	0.006	89 (76.0)	108 (59.0)	0.002
Number of stressful life events in the preceding 12 months (%)										
None	103 (34.3)	26 (24.3)	77 (39.9)		8 (17.0)	95 (37.5)		28 (23.9)	75 (41.0)	0.005
One	102 (34.0)	38 (35.5)	64 (33.2)		17 (36.2)	85 (33.6)		43 (36.8)	59 (32.2)	
Two	48 (16.0)	17 (15.9)	31 (16.1)		8 (17.0)	40 (15.8)		18 (15.3)	30 (16.4)	
Three	29 (9.7)	14 (13.1)	15 (7.8)		7 (14.9)	22 (8.7)		14 (12.0)	15 (8.2)	
Four	9 (3.0)	7 (6.5)	2 (1.0)		3 (6.4)	6 (2.4)		8 (6.8)	1 (0.5)	
Five	5 (1.7)	2 (1.9)	3 (1.6)		2 (4.3)	3 (1.2)		3 (2.6)	2 (1.1)	
Six	0 (0)	0 (0)	0 (0)		0 (0)	0 (0)		0 (0)	0 (0)	
Seven	1 (0.3)	1 (0.9)	0 (0)		1 (2.1)	0 (0)		1 (0.9)	0 (0)	
Eight	3 (1.0)	2 (1.9)	1 (0.5)	0.017	1 (2.1)	2 (0.8)	0.017	2 (1.7)	1 (0.5)	
Duration of symptoms prior to diagnosis (%)										
< 1 month	17 (5.7)	4 (3.7)	13 (6.7)		2 (4.3)	15 (5.9)		5 (4.3)	12 (6.6)	
1–3 months	95 (31.7)	25 (23.4)	70 (36.2)		12 (25.5)	83 (32.8)		29 (24.8)	66 (36.1)	
4–6 months	60 (20.0)	16 (15.0)	44 (22.8)		8 (17.0)	52 (20.6)		18 (15.4)	42 (23.0)	
7–12 months	40 (13.5)	23 (21.5)	17 (8.8)		10 (21.3)	30 (11.9)		25 (21.4)	15 (8.2)	
> 12 months	31 (10.3)	12 (11.2)	19 (9.8)		4 (8.5)	27 (10.7)		12 (10.3)	19 (10.4)	
> 24 months	57 (19.0)	27 (25.2)	30 (15.5)	0.002	11 (23.4)	46 (18.2)	0.48	28 (23.9)	29 (15.8)	0.004
Symptoms > 6 months prior to diagnosis	128 (42.7)	62 (57.9)	66 (34.2)	< 0.001	25 (53.2)	103 (40.7)	0.11	65 (55.6)	63 (34.4)	< 0.001
Prior diagnosis of anxiety or depression	48 (16.0)	33 (30.8)	15 (7.8)	< 0.001	15 (31.9)	33 (13.0)	0.001	36 (30.8)	12 (6.6)	< 0.001

* Independent samples *t*‐test for comparison of normally distributed continuous data and *χ* ^2^ for comparison of categorical data between groups.

### 3.1 Presence of Symptoms or a Formal Diagnosis of a Common Mental Disorder

Symptoms of anxiety were reported by 107 (35.7%) participants, and symptoms of depression by 47 (15.7%). Overall, 117 (39.0%) participants reported symptoms of either anxiety or depression. There was no significant difference according to type of IBD. Of the 99 patients with CD, the prevalence of symptoms of anxiety and depression were similar, with symptoms of anxiety reported by 41 (41.1%), symptoms of depression by 16 (16.2%) and symptoms of either anxiety or depression in 43 (43.4%). Similarly for the 201 participants with UC or IBD‐U, 66 (32.8%) reported symptoms of anxiety, and 31 (15.4%) symptoms of depression, with 74 (36.8%) participants reporting symptoms of either anxiety or depression. There were 22 patients (7.3%) with an existing formal diagnosis of anxiety and 39 (13.0%) with an existing formal diagnosis of depression. Of the 107 participants with symptoms of anxiety, 18 (16.8%) had an existing formal diagnosis of anxiety, and among 47 patients with symptoms of depression, 11 (23.4%) had an existing formal diagnosis of depression.

### 3.2 Factors Associated With Symptoms of a Common Mental Disorder

Patients with symptoms of a common mental disorder were significantly more likely to be younger, female and consume tobacco. No disease factors were associated with an increased likelihood of reporting symptoms of either anxiety or depression. However, a longer duration of symptoms prior to a diagnosis of IBD, higher levels of gastrointestinal symptom‐specific anxiety, and the presence of one or more stressful life events in the preceding 12‐month period were also associated with a significantly increased likelihood of reporting these symptoms (Table 1). Furthermore, an increasing number of stressful life events in the 12 months prior to diagnosis was significantly associated with the reporting of symptoms of either anxiety or depression (Table 1). Participants with CD and symptoms of a common mental disorder were again significantly more likely to be younger and have higher levels of gastrointestinal symptom‐specific anxiety (Table S1). Participants with UC or IBD‐U and symptoms of a common mental disorder were significantly more likely to have higher levels of gastrointestinal symptom‐specific anxiety and to have had a duration of symptoms prior to diagnosis of > 6 months (Table S2). Removing the 48 patients with a prior diagnosis of anxiety or depression did not significantly alter the results (Table 2).

**TABLE 2:** Characteristics of 252 patients with newly diagnosed IBD without a prior diagnosis of anxiety or depression according to the presence or absence of symptoms of anxiety or depression at baseline.

	All patients (*n* = 252)	Patients with symptoms of anxiety at baseline (*n* = 74)	Patients without symptoms of anxiety at baseline (*n* = 178)	*p* *	Patients with symptoms of depression at baseline (*n* = 32)	Patients without symptoms of depression at baseline (*n* = 220)	*p* *	Patients with symptoms of anxiety or depression at baseline (*n* = 81)	Patients without symptoms of anxiety or depression at baseline (*n* = 171)	*p* *
Mean age in years (SD)	41.7 (16.4)	36.0 (13.1)	44.1 (17.1)	< 0.001	37.2 (14.9)	42.4 (16.5)	0.098	36.5 (13.9)	44.3 (16.9)	< 0.001
Female sex (%)	112 (44.4)	41 (55.4)	71 (39.9)	0.024	16 (50.0)	96 (43.6)	0.50	44 (54.3)	68 (39.8)	0.030
Married or co‐habiting (%)	145 (57.5)	36 (48.6)	109 (61.2)	0.066	15 (46.9)	130 (59.1)	0.19	39 (48.1)	106 (70.0)	0.038
White Caucasian (%)	202 (80.2)	61 (82.4)	141 (79.2)	0.56	23 (71.9)	179 (81.4)	0.21	66 (81.5)	136 (79.5)	0.72
University graduate/professional (%)	46 (18.3)	17 (23.0)	29 (16.3)	0.211	4 (12.5)	42 (19.1)	0.37	17 (21.0)	29 (17.0)	0.44
Tobacco user (%)	34 (13.5)	15 (20.3)	19 (10.7)	0.042	9 (28.1)	25 (11.4)	0.10	17 (21.0)	17 (9.9)	0.017
Alcohol user (%)	157 (62.3)	43 (58.1)	114 (64.0)	0.38	13 (40.6)	144 (65.5)	0.007	46 (56.8)	111 (64.9)	0.21
Outpatient diagnosis (%)	236 (93.7)	70 (94.6)	166 (93.3)	0.69	28 (87.5)	208 (94.5)	0.13	74 (91.4)	162 (94.7)	0.30
IBD type (%)										
CD	86 (34.1)	30 (40.5)	56 (31.5)		12 (37.5)	74 (33.6)		32 (39.5)	54 (31.6)	
UC	133 (52.8)	34 (45.9)	99 (55.6)		16 (50.0)	117 (53.2)		38 (46.9)	95 (55.6)	
IBD‐U	33 (13.1)	10 (13.5)	23 (12.9)	0.33	4 (12.5)	29 (13.2)	0.91	11 (13.6)	22 (12.9)	0.40
Commenced 5‐aminosalicylate (%)	147 (58.3)	35 (47.3)	112 (62.9)	0.022	14 (43.8)	133 (60.5)	0.073	38 (46.9)	109 (63.7)	0.011
Commenced immunomodulator (%)	13 (5.2)	5 (6.5)	8 (4.5)	0.46	4 (12.5)	9 (4.1)	0.044	7 (8.6)	6 (3.5)	0.085
Commenced advanced therapy (%)	34 (13.5)	14 (18.9)	20 (11.2)	0.10	9 (28.1)	25 (11.4)	0.010	16 (19.8)	18 (10.5)	0.045
Commenced glucocorticosteroids (%)	84 (33.3)	32 (43.2)	52 (29.2)	0.031	15 (46.9)	69 (31.4)	0.082	35 (43.2)	49 (28.7)	0.022
Commenced any IBD‐related medication (%)	222 (88.1)	64 (86.5)	158 (88.8)	0.61	29 (90.6)	193 (87.7)	0.64	70 (86.4)	152 (88.9)	0.57
Level of gastrointestinal symptom‐specific anxiety on VSI										
Low	90 (35.7)	9 (12.2)	81 (45.5)		3 (9.4)	87 (39.5)		10 (12.3)	80 (46.8)	
Moderate	86 (69.8)	21 (28.4)	65 (36.5)	< 0.001	6 (18.8)	80 (36.4)	< 0.001	22 (27.2)	64 (37.4)	< 0.001
High	76 (30.2)	44 (59.5)	32 (18.0)		23 (71.9)	53 (24.1)		49 (60.5)	27 (15.8)	
One or more stressful life events in the preceding 12 months (%)	170 (67.5)	63 (85.1)	107 (60.1)	< 0.001	28 (87.5)	142 (64.5)	0.010	69 (85.2)	101 (59.1)	< 0.001
Symptoms > 6 months prior to diagnosis	105 (41.7)	44 (59.5)	61 (34.3)	< 0.001	17 (53.1)	88 (40.0)	0.16	46 (56.8)	59 (34.5)	< 0.001

* Independent samples *t*‐test for comparison of normally distributed continuous data and χ^2^ for comparison of categorical data between groups.

Following logistic regression, the only predictors of symptoms of a common mental disorder at the time of diagnosis were increased gastrointestinal symptom‐specific anxiety (OR 2.19; 95% CI 1.00–4.79 for moderate levels of gastrointestinal symptom specific anxiety and OR 13.5; 95% CI 5.86–31.2 for high levels of gastrointestinal symptom specific anxiety, *p* < 0.001 for trend), the presence of one or more stressful life events in the 12 months prior to diagnosis (OR 2.45; 95% CI 1.23–4.90, *p* = 0.01), and a prior diagnosis of anxiety or depression (OR 7.60; 95% CI 3.23–17.9, *p* < 0.001). Excluding the 48 patients with a prior diagnosis of anxiety or depression from the logistic regression model strengthened the findings. Increased gastrointestinal symptom‐specific anxiety (OR 2.52; 95% CI 1.05–6.10 for moderate levels of gastrointestinal symptom specific anxiety and OR 15.6; 95% CI 6.05–40.0 for high levels of gastrointestinal symptom specific anxiety, *p* < 0.001 for trend) and the presence of one or more stressful life events in the 12 months prior to diagnosis (OR 2.87; 95% CI 1.30–6.34, *p* = 0.009) remained significantly associated with the reporting of symptoms of a common mental disorder at the time of diagnosis of IBD.

## 4 Discussion

In this cross‐sectional survey we examined the prevalence and predictors of symptoms of a common mental disorder among patients newly diagnosed with IBD with objectively confirmed disease activity. Our findings demonstrate that symptoms of anxiety or depression are highly prevalent in this patient group, affecting almost 40% of patients at the time of diagnosis. The majority of patients exhibiting these symptoms at the time of their IBD diagnosis did not have a formal existing diagnosis of a common mental disorder. Disease‐related factors, such as IBD type, location, extent, or phenotype did not appear to be associated with the presence of symptoms of either anxiety or depression. Although demographic factors including female sex, younger age and tobacco consumption appear to be associated with symptoms of a common mental disorder at diagnosis, following logistic regression analysis, no baseline demographic characteristics were significantly associated with the reporting of these symptoms. The reporting of stressful life events in the 12 months prior to diagnosis and an increasing level of gastrointestinal symptom‐specific anxiety, as measured by the VSI, and a longer duration of symptoms prior to diagnosis were significantly associated with symptoms of a common mental disorder. Both the reporting of stressful life events in the 12 months prior to diagnosis and increasing levels of gastrointestinal symptom‐specific anxiety remained significant associations after logistic regression.

Eligible individuals were all identified by a single assessor, ensuring a standardised process for determining study eligibility was adhered to throughout recruitment. Being the sole provider of IBD‐related care for participants, coupled with the use of fully digitalised medical records and clinic schedules, ensures that we have included almost all eligible individuals for participation and that participants' disease characteristics are accurately recorded. Using in‐person recruitment promotes inclusivity and has resulted in over 95% of eligible individuals consenting to take part. This may, in part, have facilitated 17% of those participating being non‐White. Ethnic minorities are generally underrepresented in IBD research, despite making up almost 20% of the UK population, meaning that our findings are likely to be more representative of the UK population with IBD [27]. The inclusion of inpatients is also a strength, as these participants often have the highest gastrointestinal symptom burden and, if genuine gut‐brain effects are present in IBD, may be more likely to suffer from poor psychological health [22]. Furthermore, we recruited participants during their index presentation, when most patients were not yet well‐established on medical therapies to reduce inflammatory burden. Therefore, all were considered to have current inflammatory activity, which increases the likelihood that the effects we demonstrate represent genuine gut‐brain effects.

There are limitations of the study. We report a higher prevalence of IBD‐U than is seen among established cohorts of patients with IBD, which may affect the validity of the associations between disease‐specific factors and the presence of symptoms of a common mental disorder. However, IBD extent and phenotype often evolves with time, and our findings are in keeping with the prevalence of IBD‐U reported by others in newly diagnosed patients [28]. Endoscopies in our institution are conducted by a range of healthcare professionals across several hospital sites and, therefore, application of validated endoscopic severity scoring systems could not be mandated. Assigning endoscopic severity retrospectively based on image documentation, the quality of which may vary, could introduce bias. The inability to examine symptoms of anxiety or depression according to the degree of mucosal activity is, therefore, a potential limitation, although given all patients had endoscopic and/or radiological evidence of disease activity at the point of diagnosis, we feel this is unlikely to have impacted our findings. The multi‐site recruitment also meant it was impractical to recruit patients at the time of their diagnostic test. As such, the information and subsequent education given to patients regarding the diagnosis prior to their recruitment may vary substantially. This is of relevance, as numerous information resources are now readily accessible to patients, and although official educational resources have been demonstrated to improve anxiety levels [29], accessing alternative healthcare resources may be associated with higher anxiety [30]. Therefore, we cannot rule out that access to, or type of, information could have influenced psychological health independently prior to recruitment. Furthermore, some participants were commenced on a medical treatment at the time of their diagnosis at endoscopy, which could have improved symptom burden and, in turn, psychological health. With a mean time to recruitment of 7 days, we feel that both these factors are unlikely to have influenced our results substantially.

Inferring a diagnosis of anxiety or depression using HADS questionnaire scores may be criticised as inferior to more formal assessments of mental health, such as structured interviews. However, these questionnaires have demonstrated good specificity in patients with IBD, with the conservative cut‐offs used in our study meaning we are unlikely to have overestimated the prevalence of such symptoms [31]. In any case, the use of a structured interview would be impractical, given the large sample size. Nevertheless, we recorded whether patients had a prior existing diagnosis of anxiety or depression at the time of the initial consultation. The rates of symptom reporting we observed were in excess of the proportion of patients with a formal existing diagnosis of anxiety or depression, and most of the symptom reporting occurred in those without a diagnosis of either. In addition, the prevalence of symptoms of a common mental disorder we report in this study is similar to those using structured interviews and is, therefore, likely to be accurate [21]. Inferring a diagnosis of a common mental disorder on the basis of symptom reporting at the time of a single assessment may also be criticised. However, the stability of poor psychological health has been demonstrated previously in a large cohort of patients with IBD, with almost 90% of those with abnormal anxiety or depression scores at baseline continuing to have abnormal scores during longitudinal follow‐up [11]. Finally, despite our rigorous methodology, it is possible that some individuals meeting eligibility criteria were not recruited, for example, patients mis‐triaged to general gastroenterology clinics. We feel this is unlikely as, in our hospital, endoscopic findings suggestive of a diagnosis of IBD are triaged to dedicated specialist clinics.

The estimated prevalence of symptoms of anxiety and depression in the UK general population using HAD scores of 11 and above for each is 16.2% and 6.9% respectively [32]. Our study has identified that, for individuals newly diagnosed with IBD, the prevalence is more than double this estimate, at 35.7% and 15.7% respectively. This is comparable with the estimated prevalence of symptoms of common mental disorders in patients with an established diagnosis of IBD [33, 34, 35, 36, 37], and in line with the pooled results of a recent meta‐analysis of over 30,000 patients, where symptoms of anxiety were reported to affect more than one‐third of patients with established disease and depression more than 20% [10]. In this inception cohort we identified a higher prevalence of symptoms of both anxiety and depression than demonstrated previously in an established cohort of 1119 patients within our centre, where the prevalence of anxiety and depression was reported as 21.4% and 10.3% respectively [11]. This refutes the suggestion that the high rates of symptoms of a common mental disorder observed in IBD are merely a consequence of experiencing an adverse disease course and, in turn, supports genuine gut‐brain effects, which are more likely linked to co‐existing inflammatory burden. This is of particular relevance when considering that poor psychological health has been shown to not only influence the natural history of IBD negatively, but appears to exert a cumulative adverse effect alongside inflammatory activity [12]. Furthermore, the prevalence of a formal existing diagnosis of a common mental disorder among patients in this study is similar to that reported among the general population [32]. Stress has been implicated as a risk factor for future disease flares in IBD, predating subsequent inflammatory activity by up to 18 months [38]. Our study, similar to another inception cohort [21], has identified that prior stressful life events are highly prevalent in patients with newly diagnosed IBD, and are associated with symptoms of anxiety and depression. It is possible, therefore, that as is seen in cardiovascular disease, stress may factor in the development and progression of IBD [39]. Finally, socioeconomic status has been reported to influence psychological health, with epidemiological studies suggesting that, particularly for depression, lower socioeconomic status correlates inversely with the development of common mental disorders, and our study could therefore be criticised for its single centre design [40]. However, our findings are very similar to a multi‐centre study conducted recently in Spain [21], suggesting our results are likely to be applicable to other cohorts.

Bi‐directionality of gut‐brain interactions in IBD has been demonstrated previously [8, 9], and may confound the results of studies examining the impact of poor psychological health on future disease outcomes. Specifically, an already adverse disease course, which may then be associated with the development of symptoms of anxiety or depression, could be the main driver of a future poor prognosis, rather than symptoms of anxiety or depression driving disease activity independently. It is impossible to fully unpick the implications of poor psychological health in IBD from an assessment at one point in time. However, the current study does demonstrate that symptoms of a common mental disorder are already highly prevalent among patients with IBD at the time of their diagnosis, and prior to the development of disease‐related complications. Furthermore, most patients exhibiting these symptoms did not have a prior diagnosis of anxiety or depression, which may suggest that inflammatory activity is a true driver of poor psychological health, via gut‐brain interactions, and so may provide a plausible therapeutic target.

To our knowledge, this is the first inception cohort study to examine the relationship between symptoms of anxiety or depression and gastrointestinal symptom‐specific anxiety in IBD. Again, the association between the two supports gut‐brain effects at the time of diagnosis. Higher levels of gastrointestinal symptom‐specific anxiety have been demonstrated consistently in patients with irritable bowel syndrome, and some researchers have highlighted improvements with the use of gut‐brain neuromodulators and psychological therapies in this patient group [41, 42, 43]. A recent study demonstrated comparable reliability of the VSI in patients with IBD, which is not affected by the presence of trait anxiety, suggesting it may also represent a therapeutic target in IBD [44]. However, as most studies using the VSI are in irritable bowel syndrome, future studies examining levels of gastrointestinal symptom‐specific anxiety in patients in IBD in the context of mucosal disease activity are required to facilitate interpretation of whether these symptoms are independent of the severity of underlying inflammation.

In summary, symptoms of a common mental disorder are highly prevalent at the point of diagnosis for patients with IBD, and most occurred in patients without a formal diagnosis of anxiety or depression. They were significantly associated with gastrointestinal symptom‐specific anxiety and stressful life events occurring within the previous 12 months. To further disentangle the relationship between psychological health and disease activity and determine whether there are subsequent brain‐gut effects, longitudinal follow‐up studies of IBD inception cohorts are required to study the effects of the presence of these symptoms at diagnosis on the future course of the disease.

## Author Contributions

**Christy Riggott:** conceptualization, writing – original draft, investigation, methodology, formal analysis, project administration, data curation. **David J. Gracie:** conceptualization, investigation, writing – review and editing, methodology, supervision, formal analysis. **Alexander C. Ford:** supervision, writing – review and editing, conceptualization, investigation, methodology, formal analysis.

## Ethics Statement

This study involves human participants and was approved by the Wales research and ethics committee (REC ref.: 22/WA/0368).

## Conflicts of Interest

The authors declare no conflicts of interest.

## Supporting information


Data S1.

## Data Availability

The data that support the findings of this study are available from the corresponding author upon reasonable request.
