# Supplementary material for: Prevalence and Predictors of Symptoms of Anxiety or Depression at Diagnosis in Patients With Inflammatory Bowel Disease: An Inception Cohort
Source: Aliment Pharmacol Ther. 2025 Jun 29;62(8):788–98. doi: 10.1111/apt.70248 (PMC12453905; doi:10.1111/apt.70248)
Supplement: Supplementary file 1 — Data S1. [file APT-62-788-s001.docx]

**Supplementary Table 1. Characteristics of patients with CD according to the presence or absence of symptoms of anxiety or depression.**

|  | **All patients (n=99)** | **Patients with symptoms of anxiety at baseline**  **(n=41)** | **Patients without symptoms of anxiety at baseline**  **(n=58)** | ***p* value*** | **Patients with symptoms of depression at baseline**  **(n=16)** | **Patients without symptoms of depression at baseline**  **(n=83)** | ***p* value*** | **Patients with symptoms of anxiety or depression at baseline**  **(n=43)** | **Patients without symptoms of anxiety or depression at baseline**  **(n=56)** | ***p* value*** |
| --- | --- | --- | --- | --- | --- | --- | --- | --- | --- | --- |
| **Mean age in years (SD)** | 43.1 (17.4) | 36.8 (13.3) | 47.5 (18.6) | 0.001 | 36.3 (15.4) | 44.4 (17.5) | 0.07 | 37.4 (14.8) | 47.4 (18.1) | 0.002 |
| **Female sex (%)** | 43 (43.4) | 23 (56.1) | 20 (34.5) | 0.033 | 7 (43.8) | 36 (43.4) | 0..98 | 24 (55.8) | 19 (33.9) | 0.029 |
| **Married or co-habiting (%)** | 61 (61.6) | 21 (51.2) | 40 (70.0) | 0.074 | 6 (37.5) | 55 (66.3) | 0.030 | 22 (51.2) | 39 (69.6) | 0.06 |
| **White Caucasian (%)** | 81 (81.8) | 37 (92.0) | 44 (75.9) | 0.068 | 13 (81.3) | 68 (81.9) | 0.95 | 38 (88.4) | 43 (76.8) | 0.14 |
| **University graduate/professional (%)** | 15 (15.2) | 8 (19.5) | 7 (12.1) | 0.31 | 0 (0) | 15 (18.1) | 0.065 | 8 (18.6) | 7 (12.5) | 0.40 |
| **Tobacco user (%)** | 20 (20.2) | 10 (24.4) | 10 (17.2) | 0.38 | 7 (43.8) | 13 (15.7) | 0.010 | 11 (25.6) | 9 (16.1) | 0.24 |
| **Alcohol user (%)** | 58 (58.6) | 23 (56.1) | 35 (60.3) | 0.67 | 7 (43.8) | 51 (61.4) | 0.19 | 24 (55.8) | 34 (60.7) | 0.62 |
| **CD location (%)**  Ileal  Colonic  Ileocolonic  Ileal and upper gastrointestinal | 41 (41.1)  40 (40.4)  17 (17.2)  1 (1.0) | 17 (41.5)  16 (39.0)  8 (19.5)  0 (0) | 24 (41.4)  24 (41.4)  9 (15.5)  1 (1.7) | 0.81 | 7 (43.8)  4 (25.0)  5 (31.3)  0 (0) | 34 (41.0)  36 (43.4)  12 (14.5)  1 (1.2) | 0.32 | 19 (44.2)  16 (37.2)  8 (18.6)  0 (0) | 22 (39.3)  24 (42.9)  9 (16.1)  1 (1.8) | 0.76 |
| **Non-stricturing non-penetrating CD (%)** | 92 (92.9) | 40 (99.5) | 52 (89.7) | 0.28 | 16 (100) | 76 (91.5) | 0.48 | 42 (97.7) | 50 (89.2) | 0.24 |
| **Perianal CD (%)** | 4 (4.0) | 1 (2.4) | 3 (5.2) | 0.50 | 0 (0) | 4 (4.8) | 0.37 | 1 (2.3) | 3 (5.4) | 0.45 |
| **Commenced 5-aminosalicylate (%)** | 2 (2.0) | 0 (0) | 2 (2.4) | 0.23 | 0 (0) | 2 (2.3) | 0.53 | 0 (0) | 2 (3.6) | 0.21 |
| **Commenced immunomodulator (%)** | 11 (11.1) | 4 (9.8) | 7 (12.1) | 0.72 | 2 (12.5) | 9 (10.8) | 0.85 | 5 (11.6) | 6 (10.7) | 0.89 |
| **Commenced advanced therapy (%)** | 29 (29.3) | 12 (29.3) | 17 (29.3) | 1.00 | 6 (37.5) | 23 (27.7) | 0.43 | 13 (30.2) | 16 (28.5) | 0.86 |
| **Commenced glucocorticosteroids (%)** | 51 (51.5) | 23 (56.1) | 28 (48.3) | 0.44 | 8 (50.0) | 43 (51.8) | 0.90 | 23 (50.5) | 28 (50.0) | 0.73 |
| **Commenced any IBD-related medication(%)** | 70 (70.7) | 30 (73.2) | 40 (70.0) | 0.65 | 12 (75.0) | 58 (69.9) | 0.68 | 31 (72.1) | 39 (69.6) | 0.79 |
| **Level of gastrointestinal symptom-specific anxiety on VSI**  Low  Moderate  High | 35 (35.4)  34 (34.3)  30 (30.3) | 4 (9.8)  14 (34.1)  23 (56.1) | 31 (53.4)  20 (34.5)  7 (12.1) | <0.001 | 2 (12.5)  3 (18.8)  11 (68.8) | 33 (39.8)  31 (37.3)  19 (22.9) | 0.001 | 5 (11.6)  14 (32.6)  24 (55.8) | 30 (53.6)  20 (35.7)  6 (10.7) | <0.001 |
| **One or more stressful life events in the preceding 12 months (%)** | 68 (68.7) | 33 (80.5) | 35 (60.3) | 0.033 | 13 (81.3) | 55 (66.3) | 0.24 | 34 (79.1) | 34 (60.7) | 0.051 |
| **Number of stressful life events in the preceding 12 months (%)**  None  One  Two  Three  Four  Five  Six  Seven  Eight | 31 (31.3)  33 (33.3)  12 (12.1)  15 (15.2)  4 (4.0)  2 (2.0)  0 (0)  1 (1.0)  1 (1.0) | 8 (19.5)  14 (34.1)  5 (12.2)  8 (19.5)  4 (9.8)  0 (0)  0 (0)  1 (2.4)  1 (2.4) | 23 (39.7)  19 (32.8)  7 (12.1)  7 (12.1)  0 (0)  2 (3.4)  0 (0)  0 (0)  0 (0) | 0.053 | 3 (18.8)  4 (25.0)  3 (18.8)  4 (25.0)  1 (6.3)  0 (0)  0 (0)  1 (6.3)  0 (0) | 28 (33.7)  29 (34.9)  9 (10.8)  11 (13.3)  3 (3.6)  2 (2.4)  0 (0)  0 (0)  1 (1.2) | 0.23 | 9 (20.9)  15 (34.9)  5 (11.6)  8 (18.6)  4 (9.3)  0 (0)  0 (0)  1 (2.3)  1 (2.3) | 22 (39.3)  18 (32.1)  7 (12.5)  7 (12.5)  0 (0)  2 (3.6)  0 (0)  0 (0)  0 (0) | 0.08 |
| **Duration of symptoms prior to diagnosis (%)**  <1 month  1-3 months  4-6 months  7-12 months  >12 months  >24 months | 6 (6.1)  16 (16.2)  18 (18.2)  19 (19.2)  13 (13.1)  27 (27.3) | 2 (4.9)  8 (19.5)  4 (9.8)  12 (29.3)  3 (7.3)  12 (29.3) | 4 (6.9)  8 (13.8)  14 (24.1)  7 (12.1)  10 (17.2)  15 (25.9) | 0.11 | 2 (12.5)  1 (6.3)  3 (18.8)  4 (25.0)  2 (12.5)  4 (25.0) | 4 (4.8)  15 (18.1)  15 (18.1)  15 (18.1)  11 (13.3)  23 (27.7) | 0.72 | 3 (7.0)  8 (18.6)  4 (9.3)  13 (30.2)  3 (7.0)  12 (27.9) | 3 (5.4)  8 (14.3)  14 (25.0)  6 (10.7)  10 (17.9)  15 (26.8) | 0.06 |
| **Symptoms >6 months prior to diagnosis** | 59 (59.6) | 27 (65.9) | 32 (55.2) | 0.29 | 10 (62.5) | 49 (59.0) | 0.80 | 28 (65.1) | 31 (55.4) | 0.33 |

*Independent samples *t*-test for comparison of normally distributed continuous data and χ^2^ for comparison of categorical data between groups.

**Supplementary Table 2. Characteristics of patients with UC/IBDU according to the presence or absence of symptoms of anxiety or depression.**

|  | **All patients (n=201)** | **Patients with symptoms of anxiety at baseline**  **(n=66)** | **Patients without symptoms of anxiety at baseline**  **(n=135)** | ***p* value*** | **Patients with symptoms of depression at baseline**  **(n=31)** | **Patients without symptoms of depression at baseline**  **(n=170)** | ***p* value*** | **Patients with symptoms of anxiety or depression at baseline**  **(n=74)** | **Patients without symptoms of anxiety or depression at baseline**  **(n=127)** | ***p* value*** |
| --- | --- | --- | --- | --- | --- | --- | --- | --- | --- | --- |
| **Mean age in years (SD)** | 40.8 (15.6) | 37.6 (13.7) | 42.4 (16.2) | 0.031 | 38.5 (12.9) | 41.2 (16.0) | 0.30 | 37.7 (13.5) | 42.6 (16.4) | 0.022 |
| **Female sex (%)** | 99 (49.3) | 41 (62.1) | 58 (43.0) | 0.011 | 17 (54.8) | 82 (48.2) | 0.50 | 44 (59.5) | 55 (43.3) | 0.027 |
| **Married or co-habiting (%)** | 109 (54.2) | 32 (48.5) | 77 (57.0) | 0.25 | 15 (48.4) | 94 (55.3) | 0.48 | 36 (48.6) | 73 (57.5) | 0.23 |
| **White Caucasian (%)** | 168 (83.6) | 56 (84.8) | 112 (83.0) | 0.74 | 24 (77.4) | 144 984.7) | 0.31 | 63 (85.1) | 105 (82.7) | 0.65 |
| **University graduate/professional (%)** | 35 (17.4) | 11 (16.7) | 24 (17.8) | 0.85 | 5 (16.1) | 30 (17.6) | 0.84 | 11 (14.9) | 24 (18.9) | 0.47 |
| **Tobacco user (%)** | 25 (12.4) | 14 (21.2) | 11 (8.1) | 0.008 | 6 (19.4) | 19 (11.2) | 0.20 | 15 (20.3) | 10 (7.9) | 0.010 |
| **Alcohol user (%)** | 128 (63.7) | 40 (60.6) | 88 (65.2) | 0.53 | 14 (45.2) | 114 (67.1) | 0.020 | 43 (58.1) | 85 (66.9) | 0.21 |
| **UC/IBD-U extent (%)**  Proctitis  Left-sided  Extensive | 80 (39.8)  59 (29.4)  62 (30.8) | 25 (37.9)  18 (27.3)  23 (24.8) | 55 (40.7)  41 (30.4)  39 (28.9) | 0.69 | 11 (35.5)  10 (32.3)  10 (32.3) | 69 (40.6)  49 (28.8)  52 (30.6) | 0.86 | 28 (37.8)  20 (27.0)  26 (35.1) | 52 (40.9)  39 (30.7)  36 (28.3) | 0.60 |
| **Commenced 5-aminosalicylate (%)** | 177 (88.1) | 55 (83.3) | 122 (90.4) | 0.15 | 24 (77.4) | 153 (90.0) | 0.047 | 61 (82.4) | 116 (91.3) | 0.060 |
| **Commenced immunomodulator (%)** | 2 (1.0) | 1 (1.5) | 1 (0.7) | 0.60 | 2 (6.5) | 0 (0) | <0.001 | 2 (2.7) | 0 (0) | 0.063 |
| **Commenced advanced therapy (%)** | 10 (5.0) | 6 (9.1) | 4 (3.0) | 0.061 | 4 (12.9) | 6 (3.5) | 0.027 | 7 (9.5) | 3 (2.4) | 0.026 |
| **Commenced glucocorticosteroids (%)** | 44 (21.9) | 17 (25.8) | 27 (20.0) | 0.35 | 10 (32.3) | 34 (20.0) | 0.13 | 20 (27.0) | 24 (18.8) | 0.18 |
| **Commenced any IBD-related medication(%)** | 194 (96.5) | 63 (95.5) | 131 (97.0) | 0.57 | 30 (96.8) | 164 (96.5) | 0.93 | 71 (95.9) | 123 (96.9) | 0.74 |
| **Level of gastrointestinal symptom-specific anxiety on VSI**  Low  Moderate  High | 65 (32.3)  66 (32.8)  70 (34.8) | 8 (12.1)  16 (24.2)  42 (63.6) | 57 (42.2)  50 (37.0)  28 (20.7) | <0.001 | 4 (12.9)  3 (9.7)  24 (77.4) | 61 (35.9)  63 (37.1)  46 (27.1) | <0.001 | 10 (13.5)  17 (23.0)  47 (63.5) | 55 (43.3)  49 (38.6)  23 (18.1) | <0.001 |
| **One or more stressful life events in the preceding 12 months (%)** | 129 (64.2) | 48 (72.7) | 81 (60.0) | 0.077 | 26 (83.9) | 103 (60.6) | 0.013 | 55 (74.3) | 74 (58.3) | 0.022 |
| **Number of stressful life events in the preceding 12 months (%)**  None  One  Two  Three  Four  Five  Six  Seven  Eight | 72 (35.8)  69 (34.3)  36 (17.9)  14 (7.0)  5 (2.5)  3 (1.5)  0 (0)  0 (0)  2 (1.0) | 18 (27.3)  24 (36.4)  12 (18.2)  6 (9.1)  3 (4.5)  2 (3.0)  0 (0)  0 (0)  1 (1.5) | 54 (40.0)  45 (33.3)  24 (17.8)  8 (5.9)  2 (1.5)  1 (0.7)  0 (0)  0 (0)  1 (0.7) | 0.40 | 5 (16.1)  13 (41.9)  5 (16.1)  3 (9.7)  2 (6.5)  2 (6.5)  0 (0)  0 (0)  1 (3.2) | 67 (39.4)  56 (32.9)  31 (18.2)  11 (6.5)  3 (1.8)  1 (0.6)  0 (0)  0 (0)  1 (0.6) | 0.019 | 19 (25.7)  28 (37.8)  13 (17.6)  6 (8.1)  4 (5.4)  3 (4.1)  0 (0)  0 (0)  1 (1.4) | 53 (41.7)  41 (32.3)  23 (18.1)  8 (6.3)  1 (0.8)  0 (0)  0 (0)  0 (0)  1 (0.8) | 0.038 |
| **Duration of symptoms prior to diagnosis (%)**  <1 month  1-3 months  4-6 months  7-12 months  >12 months  >24 months | 11 (5.5)  79 (39.3)  42 (20.9)  41 (20.4)  18 (9.0)  30 (14.9) | 2 (3.0)  17 (25.8)  12 (18.2)  11 (16.7)  9 (13.6)  15 (22.7) | 9 (6.7))  62 (45.9)  30 (22.2)  10 (7.4)  9 (6.7)  15 (11.1) | 0.007 | 0 (0)  11 (35.5)  5 (16.1)  6 (19.4)  2 (6.5)  7 (22.6) | 11 (6.5)  66 (38.8)  37 (21.8)  15 (8.8)  16 (9.4)  23 (13.5) | 0.22 | 2 (2.7)  21 (28.4)  14 (18.9)  12 (16.2)  9 (12.2)  16 (21.6) | 9 (7.1)  58 (45.7)  28 (22.0)  9 (7.1)  9 (7.1)  14 (11.0) | 0.016 |
| **Symptoms >6 months prior to diagnosis** | 69 (34.3) | 35 (53.0) | 34 (25.2) | <0.001 | 15 (48.3) | 54 (31.8) | 0.073 | 37 (50.0) | 32 (25.2) | <0.001 |

*Independent samples *t*-test for comparison of normally distributed continuous data and χ^2^ for comparison of categorical data between groups.
